# Optical coherence tomography angiography (OCT-A) in retinitis pigmentosa and macular dystrophy patients: a retrospective study

**DOI:** 10.1007/s00417-021-05530-4

**Published:** 2022-01-04

**Authors:** Sebastian Deutsch, Albrecht Lommatzsch, Silke Weinitz, Ghazaleh Farmand, Ulrich Kellner

**Affiliations:** 1 Rare Retinal Disease Center, AugenZentrum Siegburg, MVZ Augenärztliches Diagnostik- Und Therapiezentrum Siegburg GmbH, Siegburg, Germany; 2 Department of Ophthalmology, University Hospital Düsseldorf, Moorenstrasse 5, 40225 Düsseldorf, Germany; 3 Eye Care Center at St. Franziskus Hospital Münster, Münster, Germany; 4 Achim-Wessing-Institute for Ophthalmologic Diagnostics, University Hospital Essen, Essen, Germany; 5 RetinaScience, Bonn, Germany

**Keywords:** Retinal imaging, Optical coherence tomography angiography, Retinitis pigmentosa, Macula dystrophy, Inherited retinal dystrophy

## Abstract

**Purpose:**

To evaluate macular vascular abnormalities in patients with macular dystrophies (MD) and retinitis pigmentosa (RP) through application of optical coherence tomography angiography (OCT-A).

**Methods:**

In this retrospective study, patients with MD and RP were examined by OCT-A and compared to healthy individuals. OCT-A images were analyzed regarding the diameter and surface area of the foveal avascular zone (FAZ) as well as flow (FL) in different retinal layers (superficial vascular complex (SVC), intermediate capillary complex (ICP), deep capillary complex (DCP), choriocapillaris (CC), and choroid (CD)).

**Results:**

Twenty-one patients with MD, 21 patients with RP without macular edema (RPnE), 8 patients with RP with edema (RPwE), and 41 healthy individuals were enrolled. The group of MD and RPnE patients showed none or only minor changes in FAZ. In RPwE patients, the FAZ was significantly smaller in vertical and horizontal measurements and surface area in SVC, whereas it was markedly enlarged in ICP. FL was significantly reduced compared to healthy individuals by an average of 13.2% in CD, 14.2% in CC, and 8.4% in DCP in all patient groups. In ICP, the reduction was 9.2% for RPnE and 12.7% for RPwE patients. The SVC showed reduced FL in the MD (8.1%) and RPnE (10.3%) group.

**Conclusions:**

OCT-A is a valuable tool to examine retinal vascular abnormalities in patients with MD and RP. OCT-A revealed a reduced flow in various retinal layers in MD, RPnE, and RPwE. Alterations of the FAZ were less distinct in these groups which add to the variation reported previously.

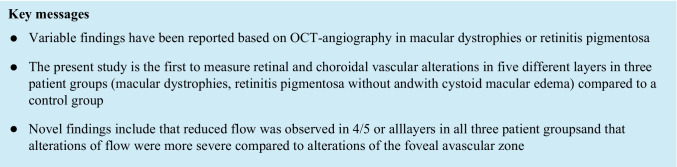

## Introduction

Inherited retinal dystrophies (IRD) are characterized by loss of visual function due to progressive degeneration of the photoreceptor/retinal pigment epithelium complex. IRD are relatively rare compared with other retinal disorders such as age-related macular degeneration, with an estimated overall frequency of approximately 1:1378 [1]. However, IRD represent the most common cause of severe visual impairment or blindness (visual acuity < 0.02 decimal or visual field residual < 5 degrees) between 21 and 60 years of age [2].

Based on the frequency of pathogenic mutations, approximately 75,000 persons are affected in Germany [2]. IRD present as a clinically and genetically very heterogeneous group of diseases with limited therapeutic options until recently. The latter is currently changing due to the development of gene therapeutic and medical treatment options [3, 4]. Therefore, early diagnosis of IRD is increasingly important to identify the natural course in early stages, to understand the pathophysiologic processes and to define the optimal time period for treatment. Non-invasive retinal imaging is crucial for establishing the diagnosis in early stages and for elaborating biomarkers that support risk determination, follow-up, and selection for therapy [5]. This study addresses the most common groups of IRD, retinitis pigmentosa (RP) [6], and macular dystrophies (MD) [7].

Mutations in about 100 genes can be associated with RP, one of the leading causes of legal blindness. Night blindness is frequently the initial sign, usually subjective impairment is due to the progressive loss of visual fields, whereas the macula frequently is involved in more progressed stages resulting in later onset of visual acuity loss [6]. Similarly, MD represents a very heterogeneous group of disorders with less associated genes but a higher variability of clinical presentation. In general, photophobia, color vision deficits, reading problems, and visual acuity loss develop early and precede visual field defects [7]. Both RP and MD may develop in association with syndromic disorders.

Though the genetic background of the majority of IRD has been identified, the details of the pathophysiologic process still need further research. Changes in the retinal vascular structures during the degenerative process of IRD are poorly understood. OCT angiography (OCT-A) is a promising diagnostic technique to analyze retinal vascular changes [8]. Here, we present alterations of the OCT-A in patients with RP or MD in comparison to a group of normal controls.

## Patients and methods

Included in this study were patients affected by IRD who underwent a clinical examination including OCT-A at the Rare Retinal Disease Center between June 2016 and June 2018 as well as volunteers from the staff included in the control group. Patients and persons of the control group were informed in detail about all examination processes and informed consent was obtained. All procedures performed in this study were in accordance with the ethical standards of the 1964 Helsinki declaration and its later amendments and approval of the local ethics board (Aerztekammer Nordrhein) was obtained.

In the patient group, 60 patients were examined:

- Retinitis pigmentosa, *n* = 31
- Macular dystrophies, *n* = 29

Patients were excluded if they had additional ocular conditions that affected or potentially affected evaluation. These include, first, opacities of the optical media (cornea, lens, vitreous) and, second, other diseases of the retina (e.g., diabetic retinopathy, hypertensive retinopathy, glaucoma, age-related macular degeneration (AMD), history of retinal detachment). In addition, all OCT-A measurements were evaluated for the results of automated segmentation and eyes without acceptable segmentation were not included in the study. In eyes with cystoid macular edema, segmentation is often arbitrary in some areas. Segmentation was considered acceptable when manual adjustment would not improve the segmentation. In 50/60 patients, at least one eye could be evaluated; in 10 patients, no sufficient data were available for evaluation due to the reasons mentioned above. To avoid additional bias in the results, only one eye from each patient was used for the analysis. This was either the eye with the available OCT-A measurement or, if OCT-A was available from both eyes, the eye that had the thinnest central retinal thickness in the OCT volume evaluation. This characteristic was used for selection because it indicated either the most severe dystrophy of the central retina or the minimal cystoid macular edema. After the initial image analysis, the group of RP patients was subdivided into patients with and without cystoid macular edema, because macular edema influenced the measured values. Patients included in the evaluation were subdivided in three groups:

- Macular dystrophies (MD), *n* = 21
- Retinitis pigmentosa, no cystoid macular edema (RPnE), *n* = 21
- Retinitis pigmentosa with cystoid macular edema (RPwE), *n* = 8

As a control population, 41 volunteers of the MVZ ADTC Siegburg GmbH were examined who had normal visual acuity and no detectable structural diseases of the eyes based on ophthalmoscopy or OCT. Both eyes were examined with OCT-A, evaluated for segmentation as described previously and the eye with the lower central retinal thickness was used for the evaluation. In the control group (CG), 41 eyes could be included.

The OCT-A were acquired with a Spectralis OCT2 (Heidelberg Engineering GmbH, Heidelberg, Germany; OCT Camera Version 1.6.5.0, Acquisition Software Version 6.7.21.0) in an examination field size of 3.2 × 3.2 mm. All images were obtained by two experienced examiners. For primary image processing and archiving, the “Heidelberg Eye Explorer” (Heidelberg Engineering GmbH, Heidelberg Germany; HEYEX version 2.4.1) with the corresponding Spectralis software version 6.12 was used. In HEYEX, the OCT-A were first evaluated regarding the quality and then measured and documented.

**Fig. 1:**
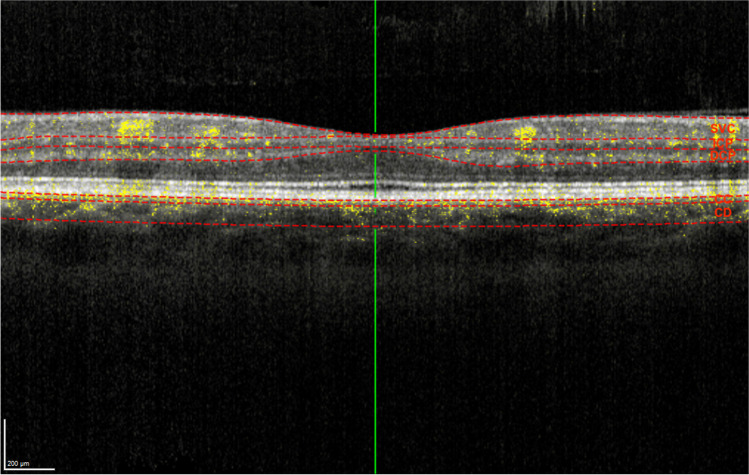
Horizontal foveal OCT b-scan in an OCT-A examination of a healthy individual. The red-dotted lines show the limits of the selected slabs, the yellow pixels represent areas where the OCT-A scan detected relevant blood flow. (SVC. superficial vascular complex; ICP, intermediate capillary plexus; DCP, deep capillary plexus; CC, choriocapillaris; CD, choroid)

After anonymization of the data, the images of all study participants were evaluated as follows: In the superficial vascular complex (SVC), intermediate capillary plexus (ICP), and deep capillary plexus (DCP), the maximum vertical and horizontal extent of the foveal avascular zone (FAZ), as well as its area, was measured manually using the tools provided by the HEYEX software. In addition, the en-face images of these layers plus the en-face images of the choriocapillaris (CC) and choroid (CD) were exported in.tiff format, saved, and analyzed for the corresponding flow (FL). A representative horizontal cross section to illustrate the selected slabs is shown in Fig. 1. SVC was limited by the Internal Limiting Membran (ILM) and the inner border of the Inner Plexiform Layer (IPL-), ICP by the inner (IPL-) and the outer (IPL +) borders of the IPL, and DCP by IPL + and the Outer Plexiform Layer (OPL) including the OPL. To define CC and CD, Bruch’s membrane (BM) has been used as reference layer. The inner limit of CC was 10 µm outside BM and from there 20 µm thick, whereas the inner limit of CD was 60 µm outside BM and 80 µm thick. The nomenclature and segmentation of the individual layers were taken from the provided HEYEX layering. We did not perform any adjustment of segmentation since the slab segmentation was rather precise or not significantly improvable due to secondary alterations associated with the retinal dystrophy (e.g., macular edema).

For the analysis of the flow of the different retinal layers, a novel method was developed with the help of the open-source image manipulation software “GNU Image Manipulation Program” (short GIMP) from the developer team around Peter Mattis in version 2.8.22 (https://www.gimp.org). Here, the OCT-A images exported from the HEYEX were examined with a grayscale analysis. To quantify the gray levels, the histogram function of the software was used, which allows a differentiation of 255 Gy levels, where gray level 0 corresponds to the color black and 255 to the color white. After extensive visual analysis, a grayscale value of 20 was set as the “no-flow” cut-off limit, such that all grayscale values ≤ 20 were classified as irrelevant blood flow. Thus, in this work, flow is defined as the percentage of pixels that have a grayscale value from 21 to 255.

Microsoft Excel version 16.35 (Microsoft, Redmond WA, USA) was used for primary data collection. For the subsequent qualitative and quantitative statistical analysis, as well as descriptive statistics, comparison of the different cohorts, and graphical presentation of the results, IBM SPSS Statistics software version 26 (IBM, Armonk NY, USA) was used. All patient groups were tested with the Shapiro–Wilk test for normal distribution and subsequent adjustment, and the Mann–Whitney *U*-test was used to define asymptotic significance, where *p* ≤ 0.05 was considered significant.

## Results

**Fig. 2:**
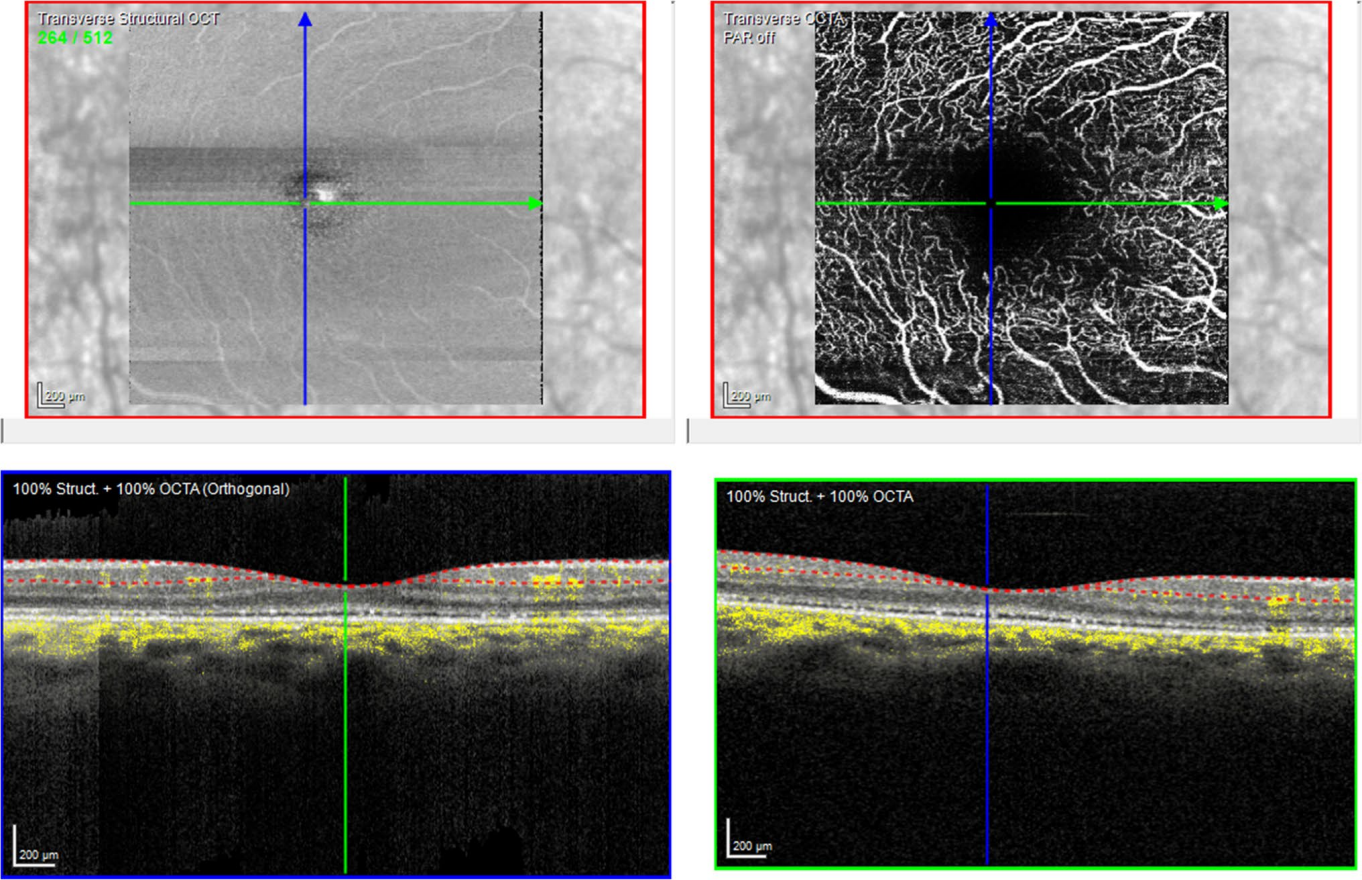
OCT-A of a patient with MD. Top left shows a transverse structural OCT scan of the selected slab (SVC) of the examined macular area (size 3.2 mm by 3.2 mm). Top right shows the OCT-A of the examined area. The vertical blue and horizontal green arrow in the top row en-face pictures correspond with the OCT B-scan in the bottom left and bottom right, which show the selected OCT-A slab marked by two dotted lines and the flow as yellow dots

**Fig. 3:**
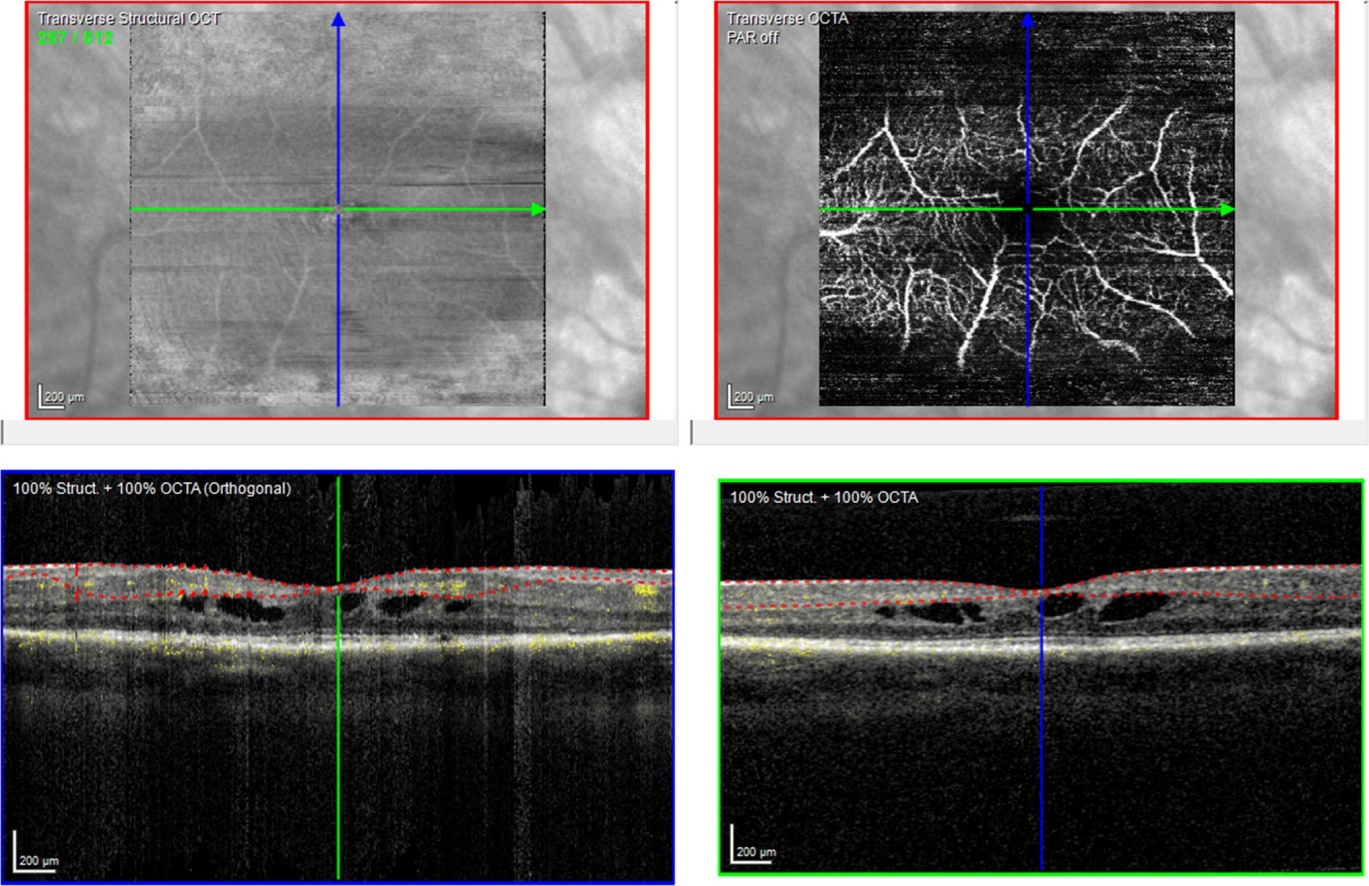
OCT-A of a RPwE patient. Top left shows a transverse structural OCT scan of the selected slab (SVC) of the examined macular area (size 3.2 mm by 3.2 mm). Top right shows the OCT-A of the examined area. The vertical blue and horizontal green arrow in the top row en-face pictures correspond with the OCT B-scan in the bottom left and bottom right, which show the selected OCT-A slab marked by two dotted lines and the flow as yellow dots

**Fig. 4:**
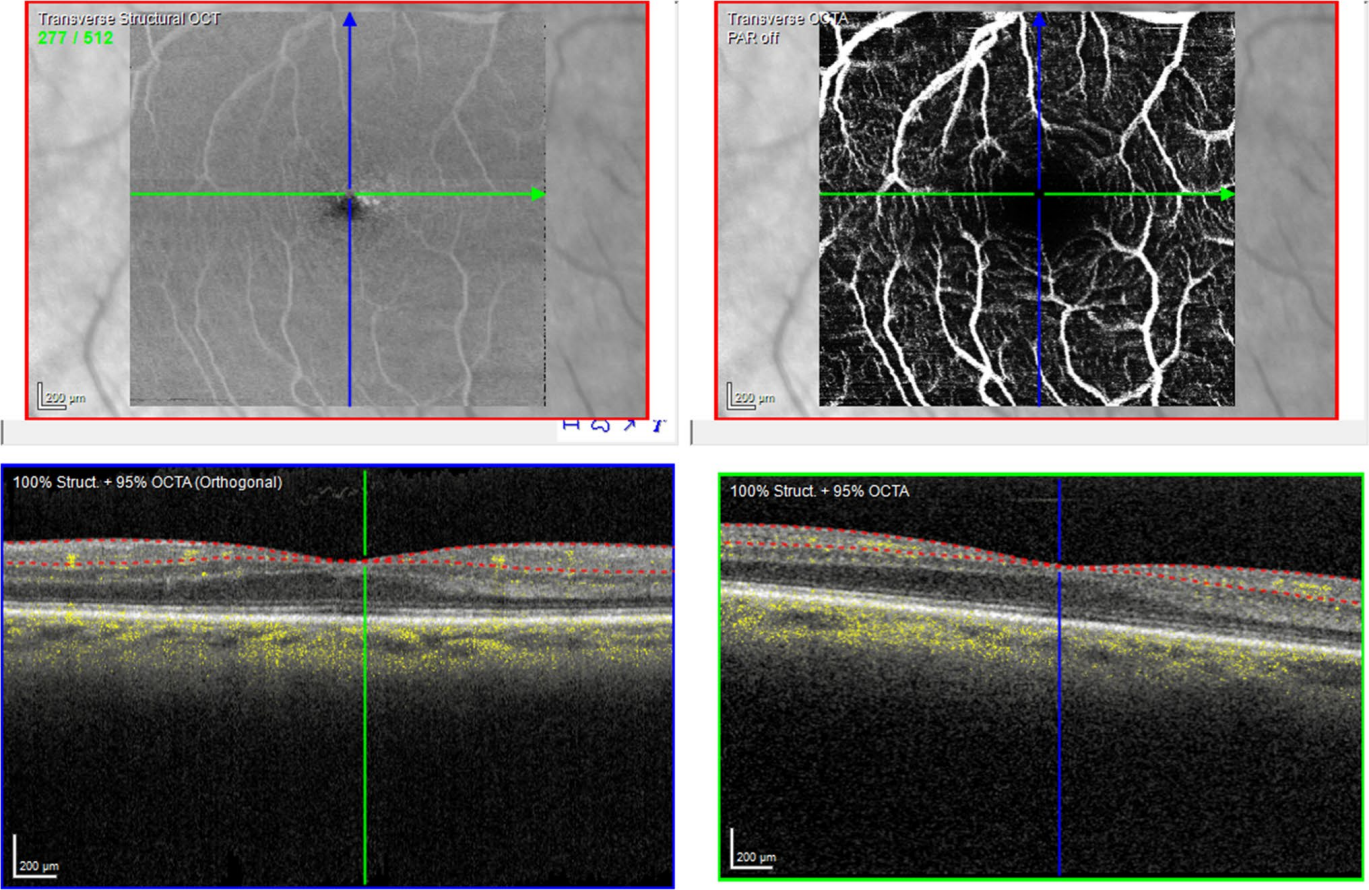
OCT-A of a RPnE patient. Top left shows a transverse structural OCT scan of the selected slab (SVC) of the examined macular area (size 3.2 mm by 3.2 mm). Top right shows the OCT-A of the examined area. The vertical blue and horizontal green arrow in the top row en-face pictures correspond with the OCT B-scan in the bottom left and bottom right, which show the selected OCT-A slab marked by two dotted lines and the flow as yellow dots

A total of 91 subjects were examined, and one eye of each subject was included in the final statistical analysis. The demographic characteristics of the patients and control subjects are shown in Table 1. The age range was comparable in all groups. In the CG, more females were included in contrast to the patient groups. Representative OCT-A scans for each disease group are shown in Figs. 2, 3, and 4.

**Table 1:** Demographic characteristics of controls and patient groups

	CG *n* = 41	MD *n* = 21	RPnE *n* = 21	RPwE *n* = 8
Sex				
Male	14	10	8	3
Female	27	11	13	5
Age (years)				
Minimum	19	17	21	23
Maximum	67	69	77	81
X̅	42.7	45.9	47.5	45.8
Eye examined				
Right	19	9	11	4
Left	22	12	10	4

*CG*, control group; *MD*, macular dystrophy; *RPnE*, retinitis pigmentosa no edema; *RPwE*, retinitis pigmentosa with edema; *X̅*, mean age

### Foveal avascular zone

**Fig. 5:**
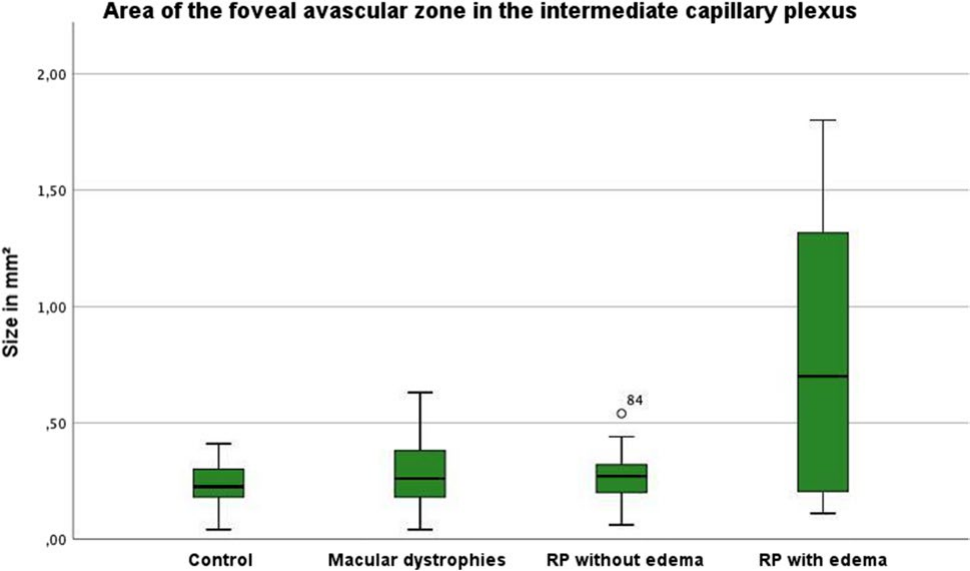
Variation of the area of the foveal avascular zone (FAZ) in the intermediate capillary plexus (ICP). Extrema deviating more than 1.5 times but less than 3 times the interquartile range (IQR) from the box are marked with a dot as potential outliers

The analysis of the FAZ showed different results for the different groups (Table 2, Fig. 5). Compared to the CG, the vertical and horizontal extent as well as the area of the FAZ was not significantly changed in the MD group in all three layers. In the RPnE group, the maximum vertical extent of the FAZ was significantly reduced (585 µm (RPnE) vs. 658 µm (CG); *p* = 0.045) in the SVC layer, and there was a trend of reduction in the DCP layer, whereas the horizontal extent and the area of the FAZ were not changed significantly in any layer.

**Table 2:** Results of the statistical analysis of the FAZ by patient groups and layers

	CG mean (95%-CI)	MD mean (95%-CI; *p*-value)	RPnE mean (95%-CI; *p*-value)	RPwE mean (95%-CI; *p*-value)
**SVC** ver	658 (CI 621 − 696)	688 (CI, 603 − 773; *p* = .447)	585 (CI, 514 − 656; *p* = **.045**)	427 (CI, 272 − 583; *p*** < .001**)
hor	737 (CI, 695 − 778)	746 (CI, 633 − 858; *p* = .873)	747 (CI 675 − 819; *p* = .785)	550 (CI 334 − 767; *p* = **.004**)
area	0.40 (CI 0.36 − 0.44)	0.45 (CI 0.33 − 0.56; p = .693)	0.35 (CI 0.29 − 0.42; *p* = .242)	0.23 (CI 0.07 − 0.38; *p* = **.003**)
**ICP** ver	515 (CI 475 − 555)	562 (CI 486 − 638; *p* = .223)	558 (CI 485 − 631; *p* = .351)	813 (CI 481 − 1145; *p* = .072)
hor	539 (CI 495 − 583)	588 (CI 508 − 669; *p* = .230)	590 (CI: 530 − 649; p = .170)	1079 (CI 593 − 1565; *p* = **.034**)
area	0.23 (CI 0.20 − 0.26)	0.28 (CI 0.22 − 0.35; *p* = .156)	0.27 (CI 0.22 − 0.32; *p* = .183)	0.79 (CI 0.26 − 1.33; *p* = **.042**)
**DCP** ver	736 (CI 695 − 776)	701 (CI 596 − 807; *p* = .534)	691 (CI 586 − 796; *p* = .224)	1014 (CI 627 − 1401; *p* = .134)
hor	841 (CI 799 − 884)	798 (CI 684 − 911; *p* = .460)	823 (CI: 734 − 911; *p* = .657)	1241 (CI 743 − 1739; *p* = .100)
area	0.50 (CI 0.46 − 0.55)	0.48 (CI 0.37 − 0.60; *p* = .736)	0.48 (CI: 0.35 − 0.60; *p* = .185)	1.16 (CI 0.43 − 1.90; *p* = .072)

Vertical and horizontal measurements displayed in µm, the area is measured in mm^2^. *CG*, control group; *MD*, macular dystrophy; *RPnE*, retinitis pigmentosa no edema; *RPwE*, retinitis pigmentosa with edema; *ver.*, vertical FAZ measurement; *hor.,* horizontal FAZ measurement; *area*, area of FAZ; *95%-CI*, 95% confidence interval; significant *p*-values printed in bold

In contrast, the RPwE group showed more significant changes compared to the CG. Here, the FAZ in the SVC was significantly decreased in both its vertical (427 µm vs. 658 µm; *p* < 0.001) and horizontal (550 µm vs. 737 µm; *p* = 0.004) extent as well as in its area (0.23mm^2^ vs. 0.40mm^2^; *p* = 0.003). In contrast, different significant changes were observed in the ICP: the horizontal diameter was almost twice as large as in the CG (1079 µm vs. 539 µm; *p* = 0.034) and the area of the FAZ was increased more than threefold (0.79mm^2^ vs. 0.23mm^2^; *p* = 0.042). There was a similar tendency in the DCP as in the ICP, but the changes were not significant.

### Flow analysis

**Fig. 6:**
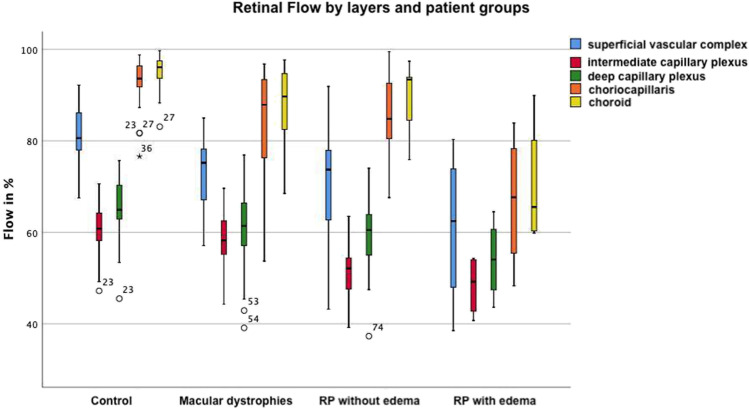
Boxplots of the flow analysis. Extrema deviating more than 1.5 times but less than 3 times the interquartile range (IQR) from the box are marked with a dot as potential outliers. Extrema deviating more than 3 times the IQR are marked as a star

Analysis of FL (expressed in %) showed significant reductions in retinal perfusion in all three patient groups in different layers (Table 3, Fig. 6).

**Table 3:** Flow density analysis

	CG mean (95%-CI)	MD mean (95%-CI; *p*-value)	RPnE mean (95%-CI; *p*-value)	RPwE mean (95%-CI; *p*-value)
SVC	80.9 (CI 78.9 − 82.9)	72.8 (CI 69.2 − 76.4; p** < .001)**	70.6 (CI 61.1 − 80.2; *p* = **.038)**	60.9 (CI 32.9 − 89.0; *p* = .107)
ICP	61.1 (CI 59.4 − 62.7)	58.3 (CI 55.6 − 61.0; *p* = .067)	51.9 (CI 48.9 − 55.1; *p*** < .001)**	48.4 (CI 37.7 − 59.1; *p*** < .001)**
DCP	65.7 (CI 63,8 − 67,5)	60.2 (CI 55.9 − 64.6; *p* = **.015)**	57.7 (CI 53.2 − 62.3; p** < .001)**	54.1 (CI 40.0 − 68.1; *p* = **.013)**
CC	93.2 (CI 91.6 − 94.7)	84.6 (CI 79.7 − 89.6; *p* = **.001)**	85.6 (CI 80.8 − 90.4; p = **.002)**	66.9 (CI 42.8 − 91.0; *p*** < .001)**
CD	95.1 (CI 94.0 − 96.2)	88.2 (CI 84.6 − 91.8; *p*** < .001)**	87.3 (CI 82.3 − 92.4; *p* = .**001)**	70.2 (CI 48.0 − 92.4; *p*** < .001)**

Values are measured in %. *CG*, control group; *MD*, macular dystrophy; *RPnE*, retinitis pigmentosa no edema; *RPwE*, retinitis pigmentosa with edema; *95%-CI*, 95% confidence interval; significant *p*-values printed in bold

Across all patient groups, the choroidal and outer retinal layers were more severely affected compared to the inner retinal layers. The mean reduction of FL in all patient groups was 13.2% in the CD and was most severe (24.9%) in the RPwE group. In the CC, the average overall reduction was even slightly more pronounced (14.2%), with the most significant reduction of 26.3% also in the RPwE group. The DCP was the most inward layer with a reduction of FL in all patient groups (8.4%), again most severe in the RPwE group. In the ICP, the RP patients showed a significant reduction for the RPnE (9.2%) and the RPwE group (12.7%), whereas the MD group did not differ from the control group. In contrast, FL in the SVC showed a significant reduction with 8.1% and 10.3% only in the MD and RPnE groups.

## Discussion

Narrowing of larger retinal vessels has been described as a characteristic sign of RP on ophthalmoscopy [6]. In contrast, large retinal vessels appear normal in MD. For both RP and MD, the knowledge about alterations of small retinal vessels in the macular area is limited. Multiple degenerative retinal disorders present changes in the avascular zone and reduction in vessel density (VD) or perfusion when examined with OCT-A [9].

Similar to previous findings, normal eyes in the CG showed some variability in the diameter and size of the FAZ. In the present study, the FAZ diameter or area did not show significant changes in any of the retinal layers examined for the MD group. Considering that macular dystrophies lead to a loss of central retinal tissue in the long run, it could be expected that the FAZ would increase concomitantly. A significant increase in the FAZ in the SVC was shown in one study for Stargardt’s disease (STGD1) patients [10]. The present series included a heterogeneous group of 21 MD patients (consisting of 7 different disorders, 3 of them with Stargardt’s disease); therefore, a direct comparison to that study is not possible. In addition, the degree of FAZ change might be variable with the degree of diseases progression included in the respective studies.

In the RPnE group, there was a significant change only for the maximum vertical FAZ diameter in the SVC, which was reduced in length, while no other changes for FAZ diameter or area were observed in all other layers. Previous studies showed that changes of the FAZ differ between studies [11–16]. A recent meta-analysis concluded that FAZ tended to be larger in SVC, but not significantly, whereas FAZ area in DCP was significantly enlarged in RP. In addition, the effects varied between SVC and DCP within the different studies, thus significant changes in FAZ could affect only the SCP, only the DCP, both layers, or neither layer. This variability can be due to the fact that FAZ changes in RP are highly variable, but it may also depend on variable inclusion criteria, as in some studies up to 50% of examined patients were not included in the evaluation due to insufficient image quality or presence of cystoid macular edema [13, 15]. Nevertheless, a reduction of the FAZ diameter as present in this study appears to be rather uncommon, even though one other study has shown a reduced FAZ area in SVC without alterations in the DCP [15].

Surprisingly, in the RPwE group, the FAZ in the SVC was significantly reduced in both its vertical and horizontal dimensions, as well as in its area. In contrast, the FAZ in the ICP was significantly enlarged, with the horizontal diameter almost twice as large as in the CG and the area of the FAZ even more increased. Changes in the DCP were similar to the ICP but not significant. It should be noted that the ICP is not separately evaluated or reported in the previous studies. The only other study comparing RPnE and RPwE patients included a rather similar number of patients but evaluated both eyes in a part of the patients [16]. They described a significant increase of FAZ area in SVC and DCP in RPwE. The difference may be due to the facts that there is already a high variability in RPnE regarding FAZ area that the correct layering is especially difficult in eyes with macular edema and that both studies included only *n* = 8 patients in the RPwE group respectively.

Perfusion analysis was the second main parameter of this study because a change in blood flow is expected in IRD based on the pathogenesis as well as on the findings from other studies [10–15, 17].

The MD group showed that there were significant reductions in FL in the SVC (*p* < 0.001), DCP (*p* = 0.015), CC (*p* = 0.001), and CD (*p* < 0.001). The reduction in FL ranged from 7% (CD) to 10% (SVC). These results are largely consistent with a previous study in which 19 Stargardt’s disease patients were presented with a reduction in retinal perfusion in SVC, DCP, and CC [10].

RPnE patients also showed a decrease in retinal perfusion. FL was significantly reduced in all five retinal layers examined, ranging from 8 (CC) to 14% (ICP). A study by Mastropasqua [17] also showed a significant reduction with respect to VD in the SCP, DCP, and the CC, so the results found here are compatible with these. For the remaining retinal layers, no comparative statements can be made in this regard.

For RPwE patients, the FL in the SVC was not significantly changed, whereas ICP, DCP, CC, and CD showed significantly decreased FL, as in the MD and RPnE group, but these were much more pronounced with reductions ranging from 17 (DCP) to 28% (CC). FL or VD have not been specifically reported for RPwE patients.

The present study contributes to the variability of OCT-A findings in IRD. Marked alterations of FL could be demonstrated for MD, RPnE, and RPwE, whereas FAZ findings in the present study parallel the heterogeneous findings in previous studies. In contrast to previous studies, more retinal layers were differentiated; as such, some of the data could not be compared to previous reports. IRD are heterogeneous, and a general limitation of this study is the heterogeneity of MD and RP disorders. In addition, the results of the RPwE are limited due to the small number of patients.

In conclusion, OCT-A is a valuable technique to detect alterations of the FAZ and especially a reduction in perfusion in IRD; however, further studies are needed to establish its clinical utility as a diagnostic tool for IRD. Gene-specific evaluation of IRD would be of high interest; however, it will be limited to the small number of patients available for studies as well as the variability of disease expression associated with a similar gene mutation.

## Data Availability

All data relevant to this study can be obtained from the corresponding author.
